# Challenges of implementing a large scale larviciding campaign against malaria in rural Burkina Faso – lessons learned and recommendations derived from the EMIRA project

**DOI:** 10.1186/s12889-016-3587-7

**Published:** 2016-09-29

**Authors:** Peter Dambach, Issouf Traoré, Achim Kaiser, Ali Sié, Rainer Sauerborn, Norbert Becker

**Affiliations:** 1 Institute of Public Health, University of Heidelberg, Heidelberg, Germany; 2 Centre de Recherche en Santé de Nouna, Nouna, Burkina Faso; 3 German Mosquito Control Association (KABS), Speyer, Germany; 4 Centre for Organismal Studies, University of Heidelberg, Heidelberg, Germany

**Keywords:** Malaria, Larviciding, Vector control, *Bacillus thuringiensis israelensis*, Burkina Faso

## Abstract

**Background:**

Recent malaria control and elimination attempts show remarkable success in several parts of sub-Saharan Africa. Vector control via larval source management represents a new and to date underrepresented approach in low income countries to further reduce malaria transmission. Although the positive impact of such campaigns on malaria incidence has been researched, there is a lack of data on which prerequisites are needed for implementing such programs on a routine basis on large scale. Our objectives are to point out important steps in implementing an anti-malaria larviciding campaign in a resource and infrastructure restraint setting and share the lessons learned from our experience during a three-year intervention study in rural Burkina Faso.

**Methods:**

We describe the approaches we followed and the challenges that have been encountered during the EMIRA project, a three-year study on the impact of environmental larviciding on vector ecology and human health. An inventory of all performed work packages and associated problems and peculiarities was assembled.

**Results:**

Key to the successful implementation of the larviciding program within a health district was the support and infrastructure from the local research center run by the government. This included availability of trained scientific personnel for local project management, data collection and analysis by medical personnel, entomologists and demographers and teams of fieldworkers for the larviciding intervention. A detailed a priori assessment of the environment and vector breeding site ecology was essential to calculate personnel requirements and the need for larvicide and application apparel. In our case of a three-year project, solid funding for the whole duration was an important issue, which restricted the number of possible donors. We found the acquisition of qualified field personnel in fair numbers not to be always easy and training in application techniques and basic entomologic knowledge required several weeks of theoretical and practical formation. A further crucial point was to establish an effective quality control system that ensured the timely verification of larviciding success and facilitated in time data handling. While the experiences of running a larviciding campaign may vary globally, the experiences gained and the methods used in the Nouna health district may be employed in similar settings.

**Conclusions:**

Our observations highlight important components and strategies that should be taken into account when planning and running a similar larviciding program against malaria in a resource limited setting. A strong local partnership, meticulous planning with the possibility of ad-hoc adaption of project components and a reliable source of funding turned out to be crucial factors to successfully accomplish such a project.

## Background

The last decade reflects a period of intense efforts in reducing malaria incidence worldwide. In Africa, recent success in malaria control has raised the debate if malaria elimination is again a goal to pursue [1–4]. Until there is no vaccine available, control measures for malaria are based on medical treatment, reduction of exposure, and vector control, mostly via Insecticide Treated Nets (ITNs) and Indoor Residual Spraying (IRS). After the era of large scale DDT spraying which ended in the 1960, to date the potential of vector larvae control in Africa is fallow. Huge potential for the control of a multitude of vector borne diseases could be reawakened with the integration of larviciding into recent control programs. Biological larvicides such as *Bacillus thuringiensis israelensis* (*Bti*) and *Bacillus sphaericus* (*Bs*) allow an environmentally sound and specific killing of vector larvae [5, 6]. Recent studies and recommendations [7] corroborate the opportunities provided by such approaches. Although not suitable for all environmental settings [8, 9], Larval Source Management (LSM) with *Bti* and *Bs* was shown efficient and cost effective in complementing existing control efforts [10–15]. Wide parts of Burkina Faso show ecosystem characteristics and climatic conditions that are eligible for running such larviciding campaigns, bearing in mind the resource scarcity of low income countries.

Apart from those studies, there is a lack of routine implementation of LSM in sub-Saharan Africa which condenses in little data on its costs and benefits on a large scale, in particular for rural West Africa. The challenge we faced, was how to setup and operationalize such a campaign in a rural health district. One of the key components of successful project management is to glean lessons from the experience gained throughout the life cycle of a project. Without a concerted effort to reflect on specific project experiences and a designated process to implement them in the running project as well as in future undertakings, lessons are lost, mistakes are repeated and opportunities for operational efficiency are missed. With this paper we want to describe and share our experience in setting up a larviciding campaign in rural Africa, point out possible stumbling blocks and try to provide a navigation aid for similar future projects.

## Methods

### Origin and visions

The EMIRA (Ecologic Malaria Reduction for Africa) root idea harks back to activities researching the vector ecology in the study region. On this basis, the decision took shape to use the gained knowledge to implement larviciding interventions embedded in a prospective intervention study on malaria transmission, infection, morbidity and mortality. A consortium of three partners (University of Heidelberg, German Mosquito Control Association (KABS) and the Nouna Health Research Center (CRSN)) was assembled in 2012, conceptualizing a project plan in the following. Based on the successful implementation of a larviciding program against nuisance floodwater mosquitoes in the Upper Rhine valley in Germany, the project was designed to nurture technology and knowledge transfer to an African context [16]. Different funding opportunities were explored, eventually deciding in favor of a private donor organization which was keen to support the transfer of larviciding technology to a developing country setting. The planning process involved sounding of expertise for the respective remits, distribution of liabilities and a first appraisal of needed material and personnel, translating into a business plan and a scientific study protocol. Upon granting, elaborate preparations for the first year in which base line data collection took place were set in motion.

The project aimed on researching the added health benefit of biological larviciding in a specific environmental setting in the Sudano-Sahelian zone of Northwestern Burkina Faso [17]. Larviciding with a *Bti* formulation (VectoBac® WG) was performed in addition to the national integrated malaria control program, which comprises the use of impregnated bed nets, intermittent preventive treatment for pregnant women (IPTp), early diagnosis and treatment of malaria cases but no indoor residual spraying (IRS). The project was a composite of different modules (see Fig. 2), including the actual larviciding intervention, the impact evaluation, and an economical assessment. Different strategies of larviciding were tested against each other and an untreated control group regarding efficacy and cost, within a health district, comprising 127 rural villages and a semi-urban town with a total of 156.000 inhabitants. Impact evaluation was performed on several levels on the connecting line between vector abundance and people’s health situation. Population health approaches provide an essential if not the most important component of impact evaluation, which is why we chose the greater Nouna region as study site, where such data are available via the Health Demographic Surveillance System (HDSS), which is part of the global INDEPTH Network [18].

### The study area

**Fig. 1:**
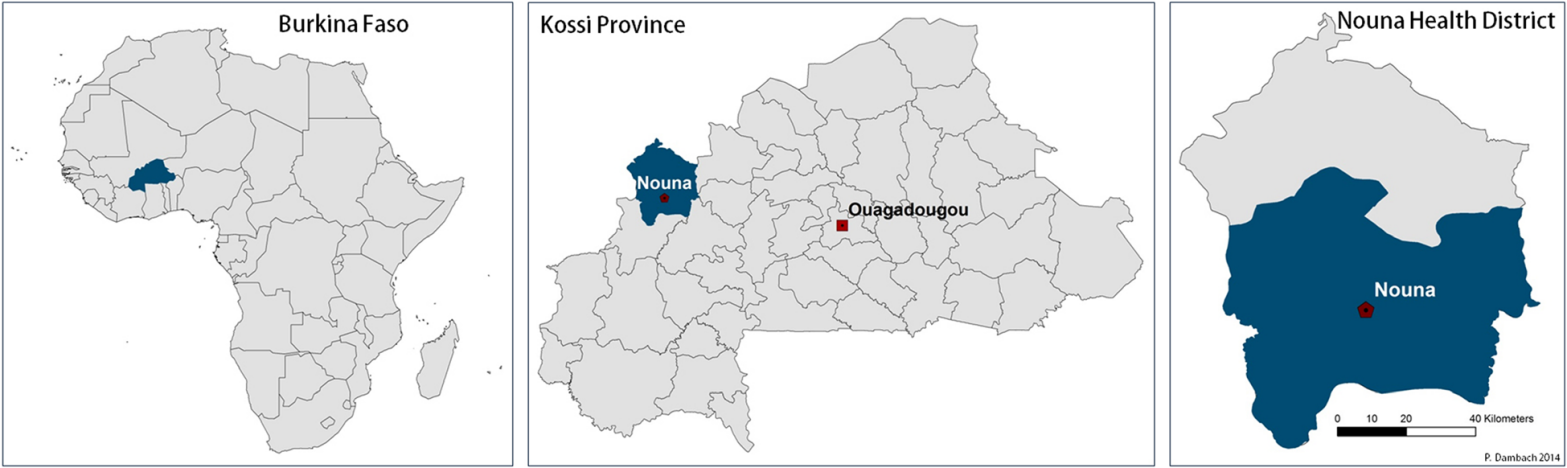
Location of Burkina Faso in Africa, the Kossi Province in North-Western Burkina Faso, and the Kossi Province with the study area in its southern part

The study area is located in the Kossi Province of northwestern Burkina Faso; the on-site base for the project was the Health Research Center (CRSN) in the town of Nouna, run by the ministry of health. The area around Nouna is rural, with most people living from subsistence farming. Malaria incidence in the region is high and shows endemicity with a marked peak during the late rainy season, which usually extends from June to September. During the rainy season large water accumulations such as rice fields, ponds, brickworks etc. develop and facilitate vector larvae breeding (Fig. 1).

### Ethical clearance and sensitization of the population

Being an amalgamation of international partners from Germany and Burkina Faso, the study had to be presented to different ethics committees. In Germany this was the ethics committee of the University of Heidelberg, and in Burkina Faso those were the national ethics board in Ouagadougou and the local committee in Nouna.

During the larviciding intervention, water bodies in villages and within a buffer of 1 km around the villages’ buildup area were treated with *Bti*. Although the intervention did not target individual households, it required information of the population. Instead of collecting individual consents, we aggregated collective informed consents for each village of intervention. Village chiefs, local mayors and responsible personnel from the health facilities were gathered in interactive meetings and received information on the project with the possibility to ask questions and express eventual concerns. A focus was put on the declarative that the larvicide used is harmless for humans, animals and the environment. Detailed information was also given on the mosquito development cycle, so that participants were able to understand the existing link between water bodies and mosquito proliferation.

### Recruitment and training of field staff

The EMIRA project as a large-scale intervention covered the area of an entire health district, comprising a total of 127 rural villages and a semi-urban town, and therefore required a plethora of contributors in different professional disciplines and at different levels of hierarchy. The formation of an operations team was regarded as an important issue. An extensive training program with different modules was defined to suit the needs of different categories of personnel. Within the project, personnel for the following tasks were needed: 1) larvicide spraying and its supervision, including basic entomologic knowledge, 2) mosquito collection for assessing vector abundance, 3) testing for parasitemia in U5 children, 4) population surveys, 5) surveys on registered disease and death cases, 6) administrative tasks.

#### Larvicide spray teams

Teams of two persons per village were recruited for the larviciding of mosquito breeding sites. Those were hired in their respective communities to raise local acceptance and benefit of their knowledge of the location of potential breeding sites. Within each village the first member of the team was the community key informant (CKI). A CKI is the person who is designated as the community health agent. She or he is the link between the community and the local rural health facility (CSPS) of reference. As community health agents, CKIs are involved in activities such as vaccinations and malaria control e.g. distribution of mosquito nets. Therefore, those are well known in their respective villages and by the district’s health authorities. The training of sprayers comprised a practical introduction to the spraying kit, the preparation of the larvicide and its correct and economical application to the breeding sites during several days in the field.

#### Spraying supervisors

The recruitment of spraying supervisors was performed via a call for tender that was published in local media by the project administration. A first selection was done based on received applications. Pre-selected candidates were invited to attend a three-day training session which was finalized by a written exam to retain the best as spraying supervisors. Non-selected candidates were put on a waiting list in order to be available in case of needed backup and reinforcing of the entomological team at place. The training had a focus on entomological aspects. In the field, supervisors had to perform a quality control of spraying activities by checking for larval densities in breeding sites one day after ensued treatment. Main points of their training were to acquire skills on dipping techniques and to distinguish mosquito larvae by genus and larval stage. Additionally, the training included the manipulation of portable GPS devices, to navigate to breeding sites.

### Material and logistics

Shipping several tons of field equipment and consumables to a rural area in Africa required not only precise planning but a considerable time safety buffer to meet project deadlines in case of delivery bottlenecks. In addition, the infrastructural prerequisites provided by the local research center were essential to allow and assure timely transport of material from the capital Ouagadougou to Nouna, which is about 300 km away. Air conditioned storage space for the larvicide had to be found and was obtained from the research center’s capacities. Attention had to be given to achieve a timely release of delivered goods at the customs.

### Adult mosquito captures

Adult mosquitoes were captured using CDC miniature light traps (John W. Hock Company, Gainesville, Florida) that were positioned in a total of 36 rural villages and the semi-urban town of Nouna. In each village 3 sample points were chosen with one outdoor and one indoor light trap each. Data collection was performed continuously, revisiting the same villages biweekly from May to November. Mosquito captures were a component which required not only specialized entomologists for vector determination but also trained field personnel for handling and maintenance of the mosquito traps.

### Collection of parasitemia data

Two rounds of testing were performed per year in a sample of U5 children (children aged under five), one during the low transmission period (December/January) and the other during the high transmission period (August/September). A total of 385 children were randomly selected in the same 36 villages in which mosquito captures were performed. The field team took blood slides and administered the individual informed consents in the local language with the help of the CKIs.

### Perception and acceptability survey

As a further instrument for assessing the intervention success and the general project acceptability a population survey was administered, driven by the positive feedback from village inhabitants reporting reduced mosquito abundance. Questionnaires with closed and open questions were used, additionally, focus group discussions were held with randomly selected participants.

### Data on malaria morbidity and mortality collected by the HDSS

**Fig. 2:**
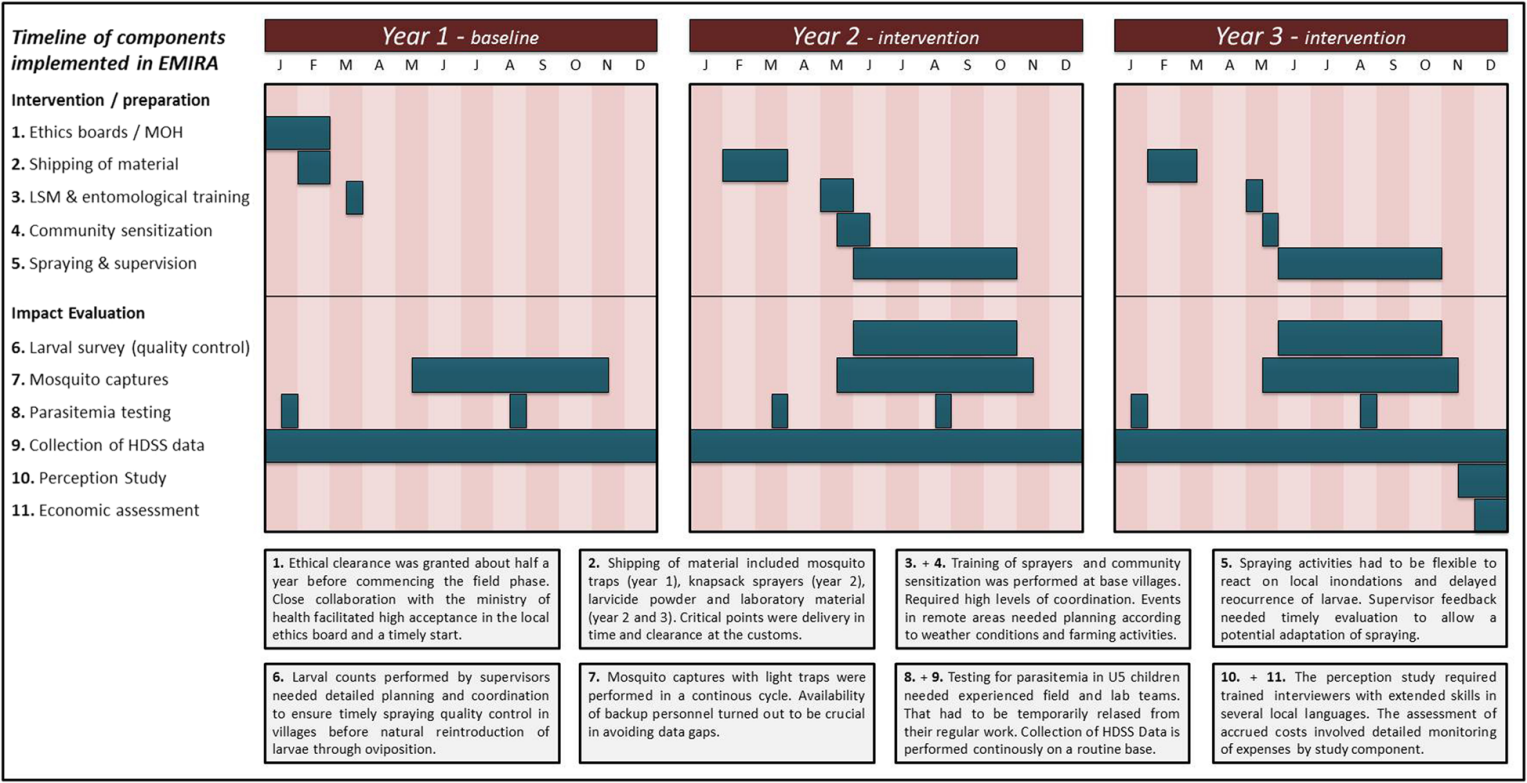
Timeline of main project components that were implemented within the EMIRA project

Malaria morbidity and mortality data constitute another way to catch the impact of vector control methods on human health. In the context of the Nouna region, it was sometimes hard to register malaria morbidity and mortality cases in an exhaustive manner since traditional medicine occupies the first place of the therapeutic itinerary, and deaths due to malaria often occur at home. CKIs were trained to collect each case of death occurring in their communities and research the cause of death by relying on verbal autopsies in areas where no HDSS data was available (Fig. 2).

## Results and discussion

### Project implementation

The consultation and involvement of project stakeholders and national authorities is a crucial prerequisite for successful project implementation. Close collaboration between research and political partners increases acceptance and facilitates promotion and ethical clearance. Furthermore, chances for future routine implementation are higher if guidelines have been developed and were coordinated together with national or international public health authorities. On the other hand, exhaustive involvement of all desired parties is not always easy. Time constraints and particularly the remote and rural research area did not facilitate regular meetings with all stakeholders at the site. Major parts of coordination and information had to be done via conference calls or during visits of the site leader at the ministry of health.

The close collaboration and high project acceptance in the field profited from the nearly two decades of ongoing research and aid projects within the rural communities. Furthermore, the strong traditional leadership system of local village chiefs facilitated information and implementation. Cultural and religious particularities that at first sight seemed to pose restrictions for successful larviciding, e.g. ponds that are considered holy and are not supposed to be altered by human activities, were discussed with traditional chiefs. Most of the alleged problems were resolved in consultation with local customs and beliefs. Those agreements required qualified personnel, high cultural sensitivity and several month of preparation time.

### Budget requirements

During the first 2 years of the project, budget requirements exceeded the initially estimated costs in several work packages. First and foremost, salaries for spray teams and supervisors had to be increased. In particular, the trans-regional and more open recruitment scheme for supervisors applied in the first year led to an unwillingness of being paid on a daily basis. People who came particularly for this job to Nouna agreed only to a monthly salary which was equivalent to comparable jobs in the public sector. During the first year of intervention it showed that significantly more on-site training and summoning of fieldworkers to the research center than planned was necessary, which equally found expression in increased salaries and allowances. For several activities such as testing for parasitemia and adult mosquito collections we realized that an increased number of team members led to higher productivity and better data quality for various reasons. Besides the team-internal control function, tasks were split and could be handled more efficiently, e.g. simultaneous taking of blood samples, performing of interviews and on site medication. Supply shortage of *Bti* larvicide powder made it necessary to ship via airfreight, which rose costs significantly. Those additional expenditures had to be synchronized with available financial assets such as overheads and imponderables.

### Recruitment and motivation of staff

While the spray teams were ideally chosen from within the respective village community in which they would work, the requirements for supervisors included a higher educational level, such as reading and writing in French language and an in depth understanding of the project components they were involved in. In Nouna itself, a first round of personnel acquisition did not deliver the desired success so that the recruitment period had to be extended. For the other areas of responsibility, the recruitment process was considerably easier and often it was possible to rely on staff from the research center that was already well trained in its respective field of work. Already established personnel structures largely facilitated the smooth flow of activities. The collection and determination of mosquitoes required entomologically trained personnel, which was available at the research center. For similar settings there might be the need for external recruitment of such personnel. We experienced that during the months of data collection and the near-term analysis workload for the two entomologic technicians and their co-workers was very high. For future activities recommendations would allow for at least one additional entomologic team or a decrease in sample locations. It turned out that in contrast to the availability of medical doctors and public health personnel, specialists or skilled technicians in entomology are difficult to find and represent possible bottlenecks in similar projects.

### Community mobilization and acceptance

Close collaboration of rural communities with the research center and vice versa and constant and detailed information delivered about the project guaranteed high acceptance amongst the population. On site visits of a communications officer and radio broadcasts led to increased project visibility and high personal identification with the project goals. During several events, the local population was gathered by the village chiefs and the project approach, methods, harmlessness of the product and the need for its meticulous application were explained. General interest and participation turned out to be higher than anticipated and venues had to be adapted or changed to accommodate for the high number of people. High project visibility, population involvement and successful vector reduction rose interest for receiving the same intervention in study arms with no and selective larviciding performed. Although the three arm study design was backed up by all three ethics committees, there was constant need for explaining the reasons for not covering all villages by the spraying intervention.

### Project success and workflow improvements

Not all data collected during the project duration have been analyzed yet but some results are already available for the achieved mosquito reductions and the malarial situation in U5 children. Compared to the baseline year, in 2014 the number of female *Anopheles* spp. was reduced by 72 % in villages where spraying of all breeding sites was performed. In the following year for the same villages, the reduction attained nearly 80 % (Dambach, unpublished data). Since there were generally higher numbers of Anopheles in 2015 compared to 2014, this additionally achieved reduction might be the fruit of improved workflow with better trained and coordinated spray teams. Although mosquito captures are not the only and ultimately desired impact evaluation parameter, they can serve as a good proxy for epidemiological impact. Future data on parasitemia, mortality, and perception will be analyzed as soon as they become available and will shed more light on the achieved impact on human health.

## Conclusions

Our vision of implementing and running a larviciding program in a health district comprising 127 villages and a semi urban town was an important step for proving the method feasible in such an environment. Although to date not all data are gathered and its analysis will be part of another research paper, a lot was learned regarding implementation and feasibility. This very large scale setting raised immense challenges due to its sheer size. An essential prerequisite for successfully running this large scale larviciding program was the timely coordination of planned activities with the ministry of health, the local research center and the national and local ethics boards. At such scale and with the goal of testing for feasibility and impact of a future routine implementation, information and involvement of political players from the health sector was of high importance. On the level of field operations planning was much more subject to short dated changes. Although we set up well-structured and detailed plans for all areas of operation, including budget, material acquisition, staff recruitment and training, larviciding and impact evaluation parameter collection, our experience showed that those activities needed constant adaptation. Imponderability mostly arose from infrastructure, e.g. the timely availability of trained staff and exceeding of delivery dates, which in the following showed influence on the planned dates of data collection. Backup personnel as well as backup dates for field activities turned out to be a crucial component of fieldwork success. Furthermore, weather events such as heavy rainfall sometimes impeded village accessibility and performing of spraying activities. A strong partnership with the local research center turned out to be a key component of success and would be favorable for the implementation of similar programs. Obtaining approval and support by the ministry of health as well as the recruitment of senior staff were highly facilitated by this close cooperation. We hope that the experiences published here will provide opportunities to successful implement and run similar programs and facilitate the circumnavigation of possible pitfalls. Our results might serve as a checklist for the implementation and improvement of resembling programs and generate evidence on feasibility for acquisition of funding from donor organizations.

## Abbreviations

Bs: Bacillus sphaericus
Bti: Bacillus thuringiensis israelensis
CDC: Center for Disease Control
CKI: Community key informant
CRSN: Centre de Récherche en Santé de Nouna
CSPS: Centre de Santé et Promotion Sociale
DDT: Dichlordiphenyltrichlorethan
EMIRA: Ecologic Malaria Reduction for Africa
IPTp: Intermittent preventive treatment for pregnant women
IRS: Indoor Residual Spraying
ITN: Insecticide Treated Nets
KABS: Kommunale Arbeitsgemeinschaft zur Bekämpfung der Schnakenplage
LSM: Larval Source Management
U5 children: Children with an age below five years
